# Heart rate response to graded exercise test of elderly subjects in different ranges of TSH levels

**DOI:** 10.20945/2359-3997000000083

**Published:** 2018-10-01

**Authors:** Rafael Cavalcante Carvalho, Patrícia dos Santos Vigário, Dhiãnah Santini de Oliveira Chachamovitz, Diego Henrique da Silva Silvestre, Pablo Rodrigo de Oliveira Silva, Mario Vaisman, Patrícia de Fátima dos Santos Teixeira

**Affiliations:** 1 Universidade Federal do Rio de Janeiro Universidade Federal do Rio de Janeiro Escola de Educação Física e Desportos Laboratório de Ergoespirometria e Cineantropometria Rio de Janeiro RJ Brasil Laboratório de Ergoespirometria e Cineantropometria, Escola de Educação Física e Desportos, Universidade Federal do Rio de Janeiro (UFRJ), Rio de Janeiro, RJ, Brasil; 2 Centro Universitário Augusto Motta Centro Universitário Augusto Motta Programa de Pós-Graduação em Ciências da Reabilitação Rio de Janeiro RJ Brasil Programa de Pós-Graduação em Ciências da Reabilitação, Centro Universitário Augusto Motta (UNISUAM), Rio de Janeiro, RJ, Brasil; 3 Universidade Estácio de Sá Universidade Estácio de Sá Rio de Janeiro RJ Brasil Universidade Estácio de Sá, Rio de Janeiro, RJ, Brasil; Pesquisa Clínica Amil, CemedCare, Niterói, RJ, Brasil; Pesquisa Clínica Amil Niterói RJ Brasil; 4 Hospital Universitário Clementino Fraga Filho Serviço de Endocrinologia Rio de Janeiro RJ Brasil Serviço de Endocrinologia, Hospital Universitário Clementino Fraga Filho (HUCFF), Rio de Janeiro, RJ, Brasil

**Keywords:** Aged, thyroid hormones, heart rate, exercise

## Abstract

**Objective:**

Life expectancy is increasing worldwide and studies have been demonstrating that elevated serum thyroid stimulating hormone (TSH) concentration in elderly is associated with some better health outcomes. This elevation is somewhat physiological as aging. The aim of this study was to investigate the heart rate (HR) response during a graded exercise test and its recovery in healthy elderly, comparing subjects within serum TSH in the lower limit of reference range to those within the TSH in the upper limit.

**Subjects and methods:**

A cross-sectional study was conducted with 86 healthy elderly aged 71.5 ± 5.1 years, with serum TSH between 0.4 – 4.0 mUl/mL. The participants were divided into two groups according to TSH level: < 1.0 mUl/mL (n = 13) and ≥ 1.0 µUI/mL (n = 73). All participants performed an ergometric test on a treadmill. The HR was recorded and analyzed at rest, during exercise and during the three minutes immediately after exercise.

**Results:**

No differences were observed in relation to HR at peak of exercise (TSH < 1.0 µUI/mL: 133.9 ± 22.5 bpm *vs.* TSH ≥ 1.0 µUI/mL: 132.4 ± 21.3 bpm; *p* = 0.70) and during the first minute of recovery phase (TSH < 1.0 µUI/mL: 122.3 ± 23.1 bpm *vs.* TSH ≥ 1.0 µUI/mL: 115.7 ± 18.4 bpm *p* = 0.33). The groups also presented similar chronotropic index (TSH < 1.0 µUI/mL: 78.1 ± 30.6 *vs*. TSH ≥ 1.0 µUI/mL: 79.5 ± 26.4; *p* = 0.74).

**Conclusion:**

In this sample studied, there were no difference between lower and upper TSH level concerning HR response during rest, peak of exercise and exercise recovery.

## INTRODUCTION

Life expectancy is increasing worldwide (1), and studies focusing on the elderly population have been demonstrating that there is a positive correlation between aging and serum thyroid stimulating hormone (TSH) concentrations (2-4). For this population, higher serum TSH levels have been associated with better outcomes regarding mortality (3,5) and functionality (5-7) than those with serum TSH in the traditional “reference range.” The evidence of possible changes in the reference range for serum TSH in the elderly may be relevant not only to the upper but also the lower limit (8). It raises the idea that in the group of euthyroid elderly subjects, there might be some people with clinical signs and symptoms of subclinical hyperthyroidism (SCH), particularly in those ones with TSH near the low reference limit.

The association between SCH and increased cardiovascular risk and mortality is well established (9-11). Autonomic system alterations as vagal attenuation and sympathetic exacerbation are also described in SCH patients (12-15). Since abnormal heart rate (HR) behavior during exercise and recovery is related to autonomic dysfunction (16), cardiovascular risk and mortality (17,18), it would be adequate to study this parameter to indirectly predict the risk of cardiovascular events. Our group previously detected a high HR at rest, a low variation between HR at the peak of exercise and HR at rest and abnormal HR recovery (15) in middle-aged women with exogenous SCH.

Considering that there is a trend towards higher values of TSH in the elderly (2-4) and that this condition is associated with better health-related variables in this population (5-8), it would be important to clarify if those with low levels of TSH would present cardiovascular impairments. Furthermore, since the elderly frequently suffer from cardiovascular disease (19), the HR variation during exercise and recovery are relevant outcomes that should be investigated. Therefore, the aim of this study was to examine the HR response during a graded exercise test and recovery in healthy elderly people with serum TSH within the reference range and compare subjects with serum TSH in the lower limit of reference to those with serum TSH in the upper limit.

## SUBJECTS AND METHODS

### Study and sample

Healthy elderly subjects of both genders, aged 65 or more years old without known thyroid dysfunctions and with serum TSH in the normal reference range (0.4–4.0 mUl/mL) were included in this cross-sectional study. The exclusion criteria were: individuals who were athletes (defined as regular physical activity > 150 minutes/week, higher than 10 METs of intensity), any previous thyroid disease, hypothalamic-pituitary disease, congestive heart failure, chronic renal disease, acute respiratory disease, diabetes mellitus, stroke, chronic obstructive pulmonary disease, asthma, cancer, cirrhosis, aortic disease, pulmonary disease, peripheral vascular disease, and coronary artery disease. Subjects using corticosteroids, amiodarone, dopamine, or any drug that interferes with the levels of thyroid hormones and/or thyroid function were excluded. Subjects taking drugs that could interfere with autonomic adjustment mechanisms (beta blockers, calcium-channel blockers, and levodopa) were also excluded. Moreover, subjects who were bedridden, wheelchair users, and those who had any physical limitation that could affect the graded exercise testing were excluded.

We divided the into two groups according to serum TSH levels: the first group was composed of those with serum TSH between 0.4 and 0.9 µUI/mL (lower reference range) and the second group included those with serum TSH between 1.0 and 4.0 µUI/mL. This cutoff point was chosen by considering cohort studies with the elderly population that showed adverse health effects in elderly people with TSH < 1.0 µUI/mL (20,21).

The study was conducted at the Clementino Fraga Filho University Hospital (HUCFF) of the Federal University of Rio de Janeiro, Brazil. The local ethics committee approved the study protocol (040/11-CEP) in accordance with the ethical standards of the institutional and national research committee and with the 1964 Helsinki declaration and its later amendments or comparable ethical standards. All participants provided written consent before being enrolled in the study.

### Laboratory analysis and exercise testing

A physician obtained anamneses, performed physical examinations, measured vital signs, and reviewed the subjects’ medical records. Then, a fasting blood collection for laboratory analysis of serum TSH and free thyroxine (FT4) levels was scheduled within one week.

TSH and free thyroxine (FT4) were measured with an immunochemiluminescence assay (Immulite; Diagnostic Products Corporation, Los Angeles, CA) with normal ranges of 0.4–4.0 µUI/mL and 0.8–1.9 ng/dL, respectively, at the Laboratory of Clinical Pathology/Hormone Section of HUCFF.

The graded exercise testing was performed on a treadmill using a ramp protocol (22). Electrocardiogram CM_5_ derivation was utilized according to the Brazilian's Ergometric National Consensus (23) using electrodes silver/silver chloride (Ag/AgCl). Participants were continuously monitored before the test (rest), during exercise, and during the first three minutes of exercise recovery. Systolic and diastolic blood pressure were checked before the test, every three minutes during exercise, and in the first and third minutes of exercise recovery (mercury sphygmomanometer; Narcosul 1400-C). The HR parameters were analyzed at three moments: when the subjects’ were at rest, during exercise, and during recovery immediately after the peak of exercise. They were considered: I) HR at rest (HRrest; bpm); II) peak HR (HRpeak; bpm); III) HR in the first and third minutes of recovery (HR1^st^ min rec and HR 3^rd^ min rec, respectively; bpm); IV) the difference between HRpeak and HRrest (∆HRpeak − HR rest; bpm); V) the difference between HRpeak and HR1^st^ min (∆ HRpeak − HR1^st^ min rec; bpm). A first-minute HR recovery ≤ 12 bpm was considered abnormal (17,24); VI) the difference between HRpeak and HR3^rd^ min (∆ HRpeak − HR3^rd^ min rec; bpm); and VII) chronotropic incompetence (CI). The CI was defined as the failure to get ≥ 80% of the predicted HR reserve (CI = (HRpeak – HRrest)/((220-age) − HRrest) x 100) (25). The participants were stimulated to perform maximal exercise. Test interruption criteria were based on the American College of Sports and Medicine recommendations (23,26). The recovery was performed at 40% of the maximum velocity at peak exercise and 0% grade.

### Statistical analyses

All analyses were performed using the software SPSS 13.0 for Windows (SPSS, Inc.). Continuous variables were presented as the mean ± standard deviation (median) and categorical variables as the relative frequency. Distribution of the variables was assessed by the Kolmogorov-Smirnov test and presented nonparametric distribution. Comparisons between the groups according to TSH stratification (TSH <1.0 µUI/mL *vs.* TSH ≥ 1.0 µUI/mL) were made using the Mann–Whitney U-test for continuous variables or the Chi-square test for categorical variables. To analyze the correlation between TSH (as a continuous variable) and HR parameters at rest, at peak of exercise and during recovery after exercise, the logarithmic transformation of TSH was made. After the certification that the logarithm of TSH assumed a normal distribution through Kolmogorov-Smirnov test, the Pearson's correlation coefficient was calculated. The statistical significance was set at p < 0.05.

## RESULTS

A total of 86 participants were included in the study. Most of them were female (67.4%) and had serum TSH ≥ 1.0 µUI/mL (84.9%). The hormonal and clinical characteristics of the sample are shown in Table 1. Elderly participants with TSH < 1.0 µUI/mL and TSH ≥ 1.0 µUI/mL did not differ in terms of age, body mass index (BMI), gender, or FT4 (p > 0.05).

**Table 1:** Clinical and hormonal characteristics of elderly patients that participated in the study

	Whole group (n = 86)	TSH < 1.0 µUI/mL (n = 13)	TSH ≥ 1.0 µUI/mL (n = 73)	p-value*
Age (years)	71.5 ± 5.1 (70.5)	71.8 ± 5.1 (71.5)	71.4 ± 5.2 (70.0)	0.54
BMI (kg/m^2^)	27.3 ± 4.4 (26.3)	28.9 ± 4.7 (27.2)	27.2 ± 4.4 (26.3)	0.58
Gender (female; %)	67.4%	53.8%	69.9%	0.22†
TSH (µUI/mL)	1.9 ± 1.1 (1.6)	0.7 ± 0.1 (0.7)	2.1 ± 1.1 (1.7)	0.00*
FT4 (ng/dL)	1.1 ± 0.2 (1.1)	1.2 ± 0.2 (1.1)	1.1 ± 0.6 (1.0)	0.08*

Results are presented as mean ± standard-deviation (median) for continuous variables and as (%) for categorical variables;

* Mann-Whitney U-test for numeric and (*†*) Fisher's Exact test for categorical variables; TSH < 1.0 µUI/mL *vs.* TSH ≥ 1.0 µUI/mL; statistical significance set at p < 0.05; BMI: body mass index; TSH: thyroid stimulating hormone; FT4: free thyroxine.

The HR parameters we considered in the study are presented in Table 2. No differences were observed between elderly patients with serum TSH < 1.0 µUI/mL and TSH ≥ 1.0 µUI/mL in relation to HR during rest, at peak exercise and during recovery after exercise (all p-values > 0.05). Concerning the first minute of recovery after exercise, 46.2% of elderly patients with TSH < 1.0 µUI/mL and 31.5% of elderly patients with TSH ≥ 1.0 µUI/mL had an abnormal HRpeak recovery i.e., less than or equal to 12 bpm, with no statistical difference between the groups (p = 0.35). The mean chronotropic index of the whole group (n = 86) was 79.3 ± 26.9 (79.1)% and there were no differences according to TSH stratification [TSH < 1.0 µUI/mL = 78.1 ± 30.6 (76.6)% *vs.* TSH ≥ 1.0 µUI/mL = 79.5 ± 26.4 (79.6)%; p = 0.74].

**Table 2:** Heart rate parameters at rest, at peak of exercise and during recovery after exercise, according to TSH stratification

	Whole group (n = 86)	TSH < 1.0 µUI/mL (n = 13)	TSH ≥ 1.0 µUI/mL (n = 73)	p-value*
HR rest (bpm)	74.7 ± 13.8 (73.5)	77.3 ± 13.1 (74.0)	74.3 ± 13.9 (73.0)	0.73
HR peak (bpm)	132.6 ± 21.4 (133.5)	133.9 ± 22.5 (133.0)	132.4 ± 21.3 (134.0)	0.70
Δ HR peak-rest (bpm)	57.8 ± 21.1 (54.5)	56.5 ± 20.5 (59.0)	58.1 ± 21.4 (54.0)	0.56
HR 1^st^ min (bpm)	116.7 ± 19.2 (116.5)	122.3 ± 23.1 (122.0)	115.7 ± 18.4 (116.0)	0.33
Δ HR peak- 1^st^ min rec (bpm)	15.8 ± 11.2 (16.5)	11.5 ± 14.3 (16.0)	16.6 ± 10.5 (17.0)	0.33
HR 3^rd^ min (bpm)	104.8 ± 17.1 (104.5)	110.0 ± 20.6 (107.0)	103.8 ± 16.4 (104.0)	0.33
Δ HR peak- 3^rd^ min rec (bpm)	11.9 ± 8.5 (11.0)	12.3 ± 8.4 (12.0)	11.8 ± 8.6 (11.0)	0.87

Results are presented as mean ± standard-deviation (median);

* Mann-Whitney U-test; TSH < 1.0 µUI/mL *vs.* TSH ≥ 1.0 µUI/mL; statistical significance set at p < 0.05; TSH: thyroid stimulating hormone HR: heart rate; Δ HR: heart rate variation; min: minute; rec: recovery.

No correlations were found between the logarithm of TSH and HR parameters at rest, at peak exercise and during recovery after exercise. The results are present in Table 3.

**Table 3:** Correlation between logarithm of TSH and heart rate parameters at rest, at peak of exercise and during recovery after exercise

	Logarithm of TSH	
	Correlation coefficient	p-value*
HR rest (bpm)	-0.010	0.465
HR peak (bpm)	-0.020	0.429
Δ HR peak-rest	-0.014	0.451
HR 1^st^ min	-0.052	0.319
Δ HR peak- 1^st^ min rec	0.051	0.323

* Pearson's test; statistical significance set at p < 0.05; TSH: thyroid stimulating hormone; HR: heart rate; Δ HR: heart rate variation; min: minute; rec: recovery.

## DISCUSSION

To our knowledge, this is the first study that has investigated the relationship between TSH variation in the normal range and HR parameters in healthy elderly subjects. The main findings were that different serum TSH levels in the normal reference range did not associate with HR abnormalities during rest, exercise, or recovery.

High values of HR at rest are associated with impaired functioning of the autonomic nervous system (ANS), with exacerbation of the sympathetic nerve and decreased vagal modulation (27-29), and this can mean less variation in the difference between HRpeak and HRrest (∆HRpeak − HR rest). The variability of HR is an important mark of chronotropic incompetence (CI), defined as the failure to get ≥ 80% of the HR reserve and described as a predictor of cardiovascular mortality and CHD events (24,30). In the population, with exogenous subclinical hyperthyroidism, our group previously demonstrated values of HR compatible with CI in middle-aged women and increased resting HR (15). The CI observed in our study, however, did not demonstrate a relationship with TSH. This may be because in an elderly population there are indications of decline in parasympathetic stimulus with a decrease in HR variability (31).

Another interesting finding, reported by Magrì and cols. (32) about the difference between HRpeak and HRrest (∆HRpeak − HRrest) compatible with CI in patients with CHF, was the correlation with functional capacity and peak oxygen uptake, i.e., the smaller the ∆HRpeak–HRrest, the smaller the functional capacity classified by the New York Heart Association. It is noteworthy that despite these findings, the authors warned to not consider a cause/effect relationship. Furthermore, Vigário and cols. (15) reported CI and impaired exercise capacity with lower oxygen consumption in middle-aged women, with exogenous subclinical hyperthyroidism; however, in our study, despite the fact that the CI could be a marker of impaired exercise capacity, it did not show a correlation with TSH near the low limit of the normal reference range.

Looking for association of TSH and functional capacity in elderly people with preexisting cardiovascular disease or who are at risk to develop such a condition, Virgini and cols. (33) correlated this with quartiles of TSH. They evaluated 5,182 participants with a mean of age 75.3 years. The groups were similar for most variables, except for BMI (lower in SCH) and hypertension (higher in subclinical hypothyroidism). Functional capacity was measured using two questionnaires: the Barthel Index (a questionnaire used to assess self-care activities of daily living and an individual's level of dependence) and the Instrumental Activities of Daily Living (a questionnaire that also measures activities of daily living, but involves interaction with the physical and social environment). No association between TSH and functional capacity were observed. In addition, after a 3-year cohort, a decrease in functional capacity without association with TSH was observed. Our results are in accordance with and provide evidence for the relevance of TSH into the normal reference range as a single mark for a decline of functional capacity.

Concerning the recovery from exercise, the first minute is an important mark of vagal imbalance, in other words, it is difficult to achieve at least 12 bpm of ∆ HRpeak − HR1^st^ min recovery difference. This abnormality is associated with an altered function in ANS, also described with the predictor of cardiovascular risk and all-cause mortality, including the elderly people (17,23). In the population, with subclinical hyperthyroidism, studies demonstrated impaired functioning of ANS (8,12,14,34); however, the patients who presented that difficult, rapidly controlled HR during recovery of exercise, were not mainly for any of the two TSH groups in our sample.

Recent studies have raised the question of whether the reference limits of TSH should be reconsidered in the elderly (8,35). Elderly patients with slightly elevated serum TSH were previously categorized as subclinical hypothyroidism patients; however, nowadays they may be classified as euthyroid subjects. In addition, previous studies showed slight benefits in the elderly from a mild increase in serum TSH, even when compared with euthyroid subjects (5). But we did not find impairment of health associated with serum TSH near the low reference range, i.e., between 0.4 and 0.9 µUI/mL, and according to Fontes and cols., (36) the low reference range remains the same in different age groups with changes in the upper limit. Collet and cols., (37) observed that the higher risk of atrial fibrillation, coronary heart disease mortality, and total mortality in patients with subclinical hyperthyroidism is mainly when the serum TSH level was lower than 0.10 mIU/L on the study with 10 cohorts.

We considered the TSH cut-off point to be < 1.00 µUI/mL and ≥ 1.00 µUI/mL in view of cohort studies with elderly people that reported adverse health effects in those with serum TSH levels < 1.00 µUI/mL (20,21). Studies have been demonstrated that serum TSH levels below 1.0 µUI/mL, even in the reference range, may have negative outcomes in elderly subjects concerning depressive symptoms and risk of hip fracture (20,21). By the possible correspondence with adverse effects related to subclinical hyperthyroidism with HR impairments, the study of TSH range next to those low abnormal values seems to be justified.

Our group evaluated the effect of antithyroid drug use in order to increase serum TSH levels to the upper normal range in elderly subjects with initial TSH levels < 1.0 μIU/mL, and analyzed the cardio-respiratory effort outcomes in a cross-sectional cohort. The results did not corroborate the hypothesis that lower serum TSH levels within the normal reference range had a negative impact on quality of life or cardiopulmonary capacity during exercise in healthy elderly people. Furthermore, the intervention intending to increase serum TSH to the upper normal range did not generate significant changes in the evaluated outcomes (38).

Admitting the difficult-to-get volunteers considering the inclusion and exclusion criteria we adopted, our small sample size – mainly those who compose the group with TSH levels < 1.0 µUI/mL – is the major limitation of the study. Although the differences in the sample sizes (i.e., TSH < 1.0 = 13 participants and TSH ≥ 1.0 = 73 participants) do not affect the Mann–Whitney test properties, we recognize that the statistical power of the test diminishes as the group size becomes more unequal (39). Future analysis, including larger sample sizes of both groups, should be useful for gaining a better understating of the associations between TSH levels and HR in the elderly population. Another limitation of our study is the absence of division groups in sedentary and physically active people.

In conclusion, the results of this study did not show a relationship between TSH and HR impairments during rest, at peak exercise, or during exercise recovery in elderly people. The groups with different levels of TSH showed a similar behavior for all variables considered in the study. Further investigations with older people (> 80 years) are necessary, whereas this population presents a higher level of TSH than our sample and it can provide a different outcome.
